# A preliminary study on degenerate characteristics of lumbar and abdominal muscles in middle-aged and elderly people with varying bone mass

**DOI:** 10.1186/s12891-023-06229-9

**Published:** 2023-02-21

**Authors:** Yun Tu, Guangyu Tang, Li Li, Rui Ji, Rui Tang, Shuling Wang, Jingqi Zhu

**Affiliations:** Department of Radiology, Shanghai Tenth People’s Hospital, Tongji University School of Medicine, 301 Middle Yanchang Road, Shanghai, 200072 China

**Keywords:** Lumbar and abdominal muscles, Degeneration, Quantitative computed tomography, Bone mass, Skeletal muscular mass index

## Abstract

**Background:**

With the wide application of QCT in the clinical assessment of osteoporosis and sarcopenia, the characteristics of musculoskeletal degeneration in middle-aged and elderly people need to be further revealed. We aimed to investigate the degenerate characteristics of lumbar and abdominal muscles in middle-aged and elderly people with varying bone mass.

**Methods:**

A total of 430 patients aged 40–88 years were divided into normal, osteopenia, and osteoporosis groups according to quantitative computed tomography (QCT) criteria. The skeletal muscular mass indexes (SMIs) of five muscles [abdominal wall muscles (AWM), rectus abdominis (RA), psoas major muscle (PMM), posterior vertebral muscles (PVM), and paravertebral muscles (PM)] included in lumbar and abdominal muscles were measured by QCT. Differences in SMIs among three groups, as well as the correlation between SMIs and volumetric bone mineral density (vBMD) were analyzed. The areas under the curves (AUCs) for SMIs for prediction of low bone mass and osteoporosis were calculated.

**Results:**

In male group, SMIs of RA and PM in osteopenia group were significantly lower than those in the normal group (*P* = 0.001 and 0.023, respectively). In female group, only SMI of RA in osteopenia group was significantly lower than that in the normal group (*P* = 0.007). SMI of RA was positively correlated with vBMD with the highest coefficients in male and female groups (*r* = 0.309 and 0.444, respectively). SMIs of AWM and RA had higher AUCs varying from 0.613 to 0.737 for prediction of low bone mass and osteoporosis in both genders.

**Conclusions:**

The changes of SMIs of the lumbar and abdominal muscles in patients with varying bone mass are asynchronous. SMI of RA is expected to be a promising imaging marker for predicting abnormal bone mass.

**Trial registration:**

ChiCTR1900024511 (Registered 13–07-2019).

## Background

With the aggravation of social aging, the prevalence of musculoskeletal diseases is increasing, which has become a global public health problem [1–3]. In 2019, the revised diagnostic consensus drafted by the European Working Group on Sarcopenia in Older People (EWGSOP) defined sarcopenia as "a muscle disease that results from long-term accumulation of adverse muscle changes" [4]. In the elderly population, sarcopenia is characterized by age-related progressive, systemic muscle loss and/or muscle strength decrease or muscle physiological function decline [1]. The prevalence of sarcopenia was reported as 5–13%, while the prevalence ranged from 11 to 50% in elder population (> 80 years) [1]. A recent meta-analysis study reported that the prevalence of sarcopenia ranged from 10 to 27% using different classification systems [2]. Osteoporosis is a systemic metabolic disease related to decreased bone strength, bone microstructure destruction, and increased bone fragility [5]. Globally, the prevalence of osteoporosis was 18.3%, and it is greater in women than in men (23.1% vs. 11.7%) [3]. The relationship between sarcopenia and osteoporosis has become a research hotspot [6–8]. Revealing the relationship between muscular mass and bone mass can contribute to further understand the linkage between muscle degeneration and bone deterioration.

Presently, skeletal muscular mass and bone mass are commonly measured by dual X-ray absorbtiometry (DXA) or quantitative computed tomography (QCT) [4, 9–12]. DXA classifies organs and muscle tissue into the category of thin tissue, which is not accurate in the evaluation of abdominal muscle tissue, and can not measure a single target muscle. Although DXA is the gold standard for the diagnosis of osteoporosis, its accuracy for the measurement of areal bone mineral density (aBMD) has been widely questioned, especially in middle-aged and elderly population [12, 13]. QCT can accurately quantify volumetric BMD (vBMD) and body composition through phantom and post-processing software. Therefore, more clinical musculoskeletal studies were conducted on QCT technique [14–18]. Previous studies frequently used the muscular mass of total muscles or alone used paravertebral muscles, psoas major muscle, or posterior vertebral muscles at the middle level of lumbar 3 (L3) as the markers to represent muscular mass [14–18]. However, few studies grouped lumbar and abdominal muscles into different muscles on the midaxial level of L3 vertebral body and investigated the degenerate characteristics of different muscles in middle-aged and elderly people [4, 10, 11]. According to different classification systems, the prevalences of different genders were not certain. However, based on the different physiological characteristics of men and women, the diagnostic threshold of sarcopenia was usually lower in female subjects [2]. Whether the pattern of muscle degeneration is consistent in subjects of different genders is unknown.

This preliminary study aims to investigate the degenerate characteristics of lumbar and abdominal muscles with varying bone mass based on QCT technique in male and female middle-aged and elderly people, and the relationships between muscular masses of different muscles and bone mass are discussed as well. Here we hypothesize that the decline of muscle mass in different muscle groups maybe synchronous in middle-aged and elderly people with varying age and bone mass.

## Materials and methods

### Patient population

Between July 2019 and May 2021, a total of 445 subjects underwent lumbar QCT examination in Shanghai Tenth People’s Hospital. Inclusion criteria were as follows: 1) patient aged ≥ 40 years; and 2) volunteer to participate in lumbar QCT examination. Exclusion criteria were as follows: 1) poor quality of QCT images affected observation and measurement (such as obvious artifact, severe degenerative changes or fracture deformity, and implants, hardware, devices, or other foreign material in the measurement area); 2) individuals with bone dysplasias known to have excessive fracture risk (osteogenesis imperfecta, osteopetrosis) or high BMD (such as prolonged exposure to fluoride); 3) history of malignancy with or without treatment; 4) history of drug therapy affecting musculoskeletal metabolism more than 3 months (anti-osteoporosis drugs, sex hormone, glucocorticoids, etc.); 5) male individuals with surgically or chemotherapeutically induced castration; 6) with an endocrine disorder known to affect BMD (such as hyperparathyroidism, hyperthyroidism, growth hormone deficiency or Cushing’s syndrome); 7) individuals with medical conditions known to alter BMD (such as renal failure, arthritis, chronic bowel diseases, enteral and parenteral nutrition, cachexia or bedridden for more than 1 week within the last 3 mouths); 8) history of regular physical exercise within the past 1 year [19]. After excluding 15 cases, the current study included 430 cases [male: female = 162 (37.7%): 268 (62.3%); age, 40–88 years; mean age, (60.3 ± 8.8) years]. All participants were divided into four groups based on age (40–49, 50–59, 60–69, and ≥ 70 years). Ethics committee of Shanghai Tenth People’s Hospital approved this prospective study (Number: SHSY-IEC-4.1/18–200/01), which was also registered on Chinese Clinical Trial Registry (Number: ChiCTR1900024511). Informed consent was obtained from all participants.

### QCT examination

#### Bone mass measurement

All subjects underwent CT scan of lumbar vertebrae from L1 to L3 with a dual-source CT (Somatom Force, Siemens Healthcare, Forchheim, Germany). A solid-state CT calibration phantom (Mindways Software Inc., Austin, TX, USA) which was placed under the waist was used simultaneously during the scan. The scanning parameters were as follows: tube voltages, 120 kV; tube current, 125 mAs; slice thickness 5 mm; reconstructed slice thickness 1.5 mm; and matrix size, 512 × 512. Images were transferred to a QCT workstation and analyzed using QCT PRO 5.10 software (Mindways Software Inc., Austin, TX, USA). Regions of interest (ROI) were placed in the central part of L1–L3 vertebral bodies on axial, sagittal, and coronal images. The margin of ROI should be more than 3 mm within of the border of vertebral body to avoid partial volume effect from the cortical bone. The vBMD value was the mean vBMD of L1–L3. The subjects were divided into normal group (vBMD > 120 mg/cm^3^), osteopenia group (80 mg/cm^3^ ≤ vBMD ≤ 120 mg/cm^3^), and osteoporosis group (vBMD < 80 mg/cm^3^) according to ACR criteria [19].

### Muscular mass measurement

**Fig. 1:**
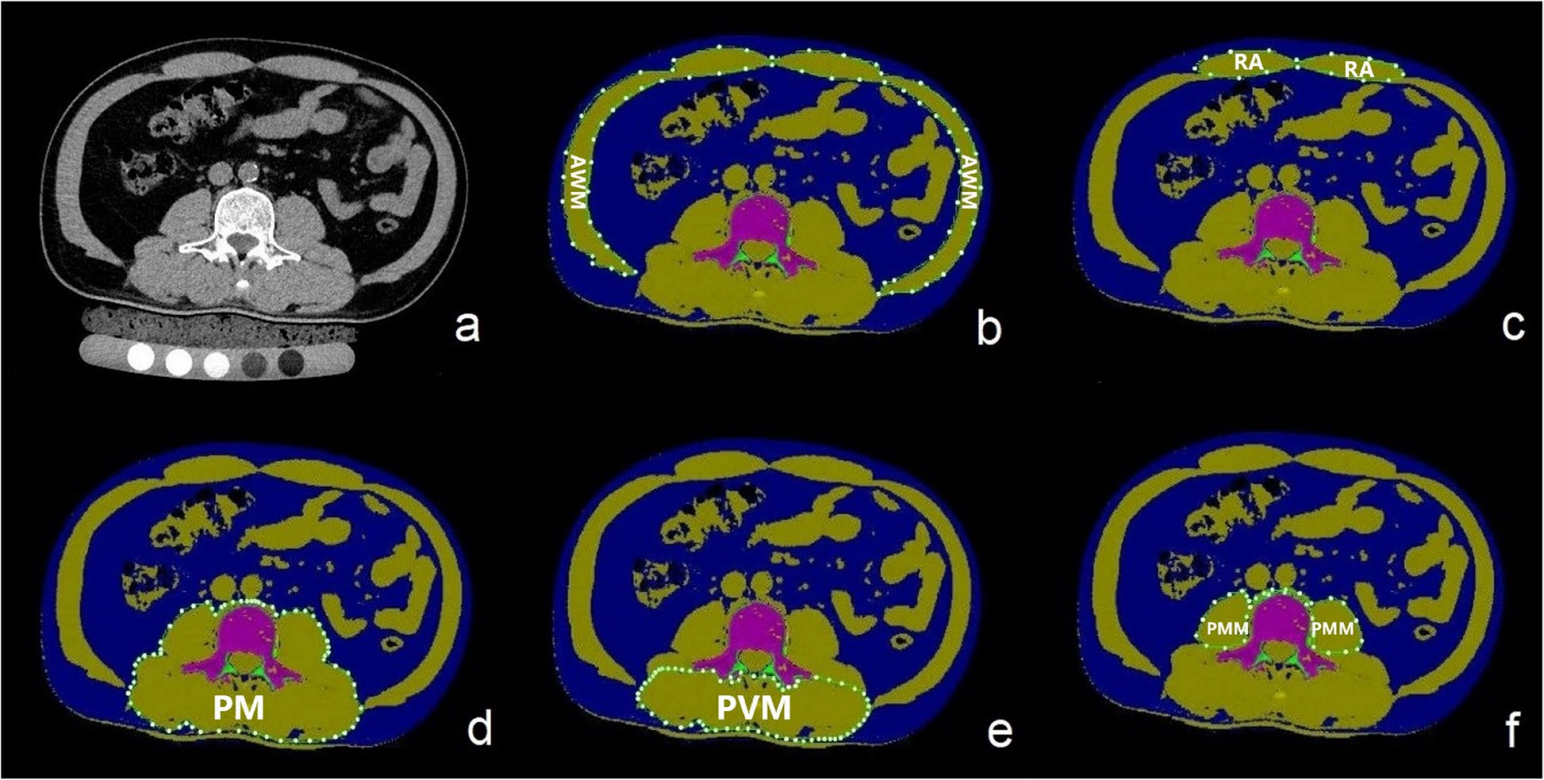
**(a)** QCT image on the midaxial level of L3 vertebral body. The ROIs of abdominal wall muscles (AWM) **(b)**, rectus abdominis (RA) **(c)**, paravertebral muscles (PM) **(d)**, posterior vertebral muscles (PVM) **(e)**, and psoas major muscle (PMM) **(f)** on the same QCT image

Body composition parameters were acquired by QCT images with 1.5 mm reconstructed slice thickness on the midaxial level of L3 vertebral body. QCT PRO 5.10 software was used for quantitative analysis of muscle composition (CT value between -29 ~ 150 HU) in the bilateral lumbar and abdominal muscles, which were divided into 5 groups: rectus abdominis (RA), posterior vertebral muscles (PVM), psoas major muscle (PMM), paravertebral muscles (PM, defined as the sum of PMM, quadratus lumborum, and PVM), and abdominal wall muscles (AWM, defined as all muscles except PM) on the midaxial level of L3 vertebral body) [11]. The cross-sectional area (CSA) of muscle was calculated automatically after the ROI drawn manually along the border of target muscle **(**Fig. 1**)**. In recent years, the main evaluation indexes of sarcopenia using QCT technique in most studies were CSA and skeletal muscular mass index [SMI; SMI = CSA (cm^2^)/ height squared (m^2^)] on the midaxial level of the L3 vertebral body [20, 21]. Therefore, SMI was selected as a more ideal evaluation indicator to calculate the muscular mass.

### Repeatability analysis

Thirty subjects including 15 males and 15 females were selected by using random number generator. Radiologists Zhu J and Tang G who had more than 15 years experience in musculoskeletal radiology were trained to draw ROI using the same method and QCT software. Neither Zhu J nor Tang G knew the clinical information of the subjects including name, gender, height, weight, history of menopause, medical history, and other factors that may affect the measurement. After one week, Zhu J repeated the measurement of the same 30 subjects. Intraclass correlation coefficient (ICC) was used to evaluate the repeatability of the two measurements of two radiologists and two measurements of the same radiologist. All the data would have been measured independently by Zhu J if good agreements (ICC ≥ 0.75) had been found.

### Statistical analysis

The analyses were performed by SPSS 25.0 (SPSS, Chicago, IL, USA). The normality analysis of continuous data was performed by the Shapiro–Wilk test. Normally distributed variables were presented as mean ± standard deviation (SD). Non-normally distributed variables were expressed as median (interquartile range). The differences of vBMD among multiple groups (four groups based on age) were analyzed by one-way ANOVA (Bonferroni; for normal variables) or Kruskal–Wallis H test (for non-normal variables). The differences of SMIs of five muscles among multiple groups (four groups based on age and three groups based on vBMD) were analyzed by one-way ANOVA (Bonferroni; for normal variables) or Kruskal–Wallis H test (for non-normal variables). The Pearson’s and Spearman’s correlation analyses were used for normal and non-normal variables, respectively. Receiver-operating characteristic (ROC) analysis was performed by Medcalc version 15.6 (MedCalc Software, Mariakerke, Belgium) to evaluate the diagnostic efficacy of SMIs of different muscles for differentiating between normal bone mass and low bone mass (including osteopenia and osteoporosis), as well as between non-osteoporosis (including normal bone mass and osteopenia) and osteoporosis. *P* value less than 0.05 was considered statistically significant.

## Results

### Repeatability evaluation

Inter-observer agreement: ICCs of vBMD and SMIs of five muscle groups indicated good agreement varying from 0.810 to 0.974.

Intra-observer agreement: ICCs of vBMD and SMIs of five muscle groups indicated good agreement varying from 0.750 to 0.973 (Table 1).

**Table 1:** Inter- and intra-observer agreement for QCT parameters

ICC (95% CI)	vBMD	SMI				
		**AWM**	**RA**	**PM**	**PVM**	**PMM**
Inter-observer	0.966 (0.929–0.984)	0.974 (0.947–0.988)	0.876 (0.757–0.939)	0.967 (0.932–0.984)	0.939 (0.876–0.970)	0.810 (0.638–0.905)
Intra-observer	0.969 (0.935–0.985)	0.959 (0.915–0.980)	0.750 (0.538–0.873)	0.973 (0.884–0.972)	0.930 (0.867–0.966)	0.820 (0.656–0.910)

*AWM* abdominal wall muscles, *CI* confidence interval, *ICC* intraclass correlation coefficient, *PM* paravertebral muscles, *PMM* psoas major muscle, *PVM* posterior vertebral muscles, *QCT* quantitative computed tomography, *RA* rectus abdominis, *SMI* skeletal muscular mass index, *vBMD* volumetric bone mineral density

### Comparison of musculoskeletal mass among varying age

For both genders, vBMD decreased continuously with age. In male group, vBMD significantly decreased (*P* = 0.008) in the third age range (60–69 years), compared with the first age range (40–49 years). In female group, vBMD significantly decreased (*P* = 0.008) in the second age range (50–59 years), compared with the first age range (40–49 years).

For both genders, SMI decreased continuously with age in all muscle groups except for the PMM. In male group, SMIs of PM and PVM significantly decreased (*P* = 0.004 and 0.016, respectively) in the second age range (50–59 years), and SMIs of AWM, RA, and PMM significantly decreased (*P* = 0.045, 0.002, and 0.002, respectively) in the third age range (60–69 years), compared with the first age range (40–49 years). In female group, SMI of RA significantly decreased (*P* = 0.013) in the second age range (50–59 years), SMI of AWM significantly decreased (*P* = 0.002) in the third age range (60–69 years), and SMI of PVM significantly decreased (*P* = 0.028) in the fourth age range (≥ 70 years), compared with the first age range (40–49 years) (Table 2).

**Table 2:** Comparison of musculoskeletal mass among varying age

Parameter	Age (male)					Age (female)				
	**40–49 years**	**50–59 years**	**60–69 years**	** ≥ 70 years**	***P* value**	**40–49 years**	**50–59 years**	**60–69 years**	** ≥ 70 years**	***P* value**
	***n* = 22**	***n* = 74**	***n* = 54**	***n* = 12**		***n* = 25**	***n* = 75**	***n* = 130**	***n* = 38**	
vBMD (mg/cm^3^)	141.76 ± 27.88	129.27 ± 30.39	115.23 ± 33.02^a^	99.99 ± 42.29^ab^	< 0.001	167.70(149.45,213.95)	123.70(103.60,146.80)^a^	88.90(71.38,108.35)^ab^	58.45(45.68,73.48)^abc^	< 0.001
AWM SMI (cm^2^/m^2^)	23.02(17.97,24.55)	20.47(17.71,22.71)	19.54(16.03,21.88)^a^	17.32(16.47,21.14)	0.014	15.82(14.53,17.29)	14.90(13.29,16.56)	13.95(12.09,15.75)^a^	12.80(10.74,14.45^)ab^	< 0.001
RA SMI (cm^2^/m^2^)	4.68 ± 1.40	3.96 ± 1.51	3.37 ± 1.32^a^	2.93 ± 1.03^a^	< 0.001	3.52 ± 1.08	2.86 ± 0.86^a^	2.45 ± 0.91^ab^	1.72 ± 0.94^abc^	< 0.001
PM SMI (cm^2^/m^2^)	31.48 ± 3.59	28.44 ± 3.77^a^	27.00 ± 3.56^a^	25.59 ± 2.17^ab^	< 0.001	23.91 ± 2.55	22.60 ± 2.73	22.31 ± 3.27	22.20 ± 4.09	0.130
PVM SMI (cm^2^/m^2^)	18.09 ± 2.31	16.38 ± 2.23^a^	15.73 ± 2.28^a^	13.90 ± 2.90^ab^	< 0.001	13.91 ± 1.90	13.77 ± 1.82	13.38 ± 2.32	12.30 ± 2.56^abc^	0.005
PMM SMI (cm^2^/m^2^)	9.59(8.62,10.88)	8.76(7.73,9.86)	8.06(6.83,8.93)^a^	8.65(6.97,8.93)	0.004	7.21(6.15,7.82)	6.48(5.41,7.41)	6.35(5.39,7.90)	7.10(5.51,11.00)	0.100

Data were expressed as mean ± standard deviation or median (interquartile range)

*AWM* abdominal wall muscles, *PM* paravertebral muscles, *PMM* psoas major muscle, *PVM* posterior vertebral muscles, *RA* rectus abdominis, *SMI* skeletal muscular mass index, *vBMD* volumetric bone mineral density

^a^ Compared with 40–49 years group, *P* < 0.05

^b^ Compared with 50–59 years group, *P* < 0.05

^c^ Compared with 60–69 years group, *P* < 0.05

### Correlation between musculoskeletal mass and age

For both genders, vBMD was negatively correlated with age (*r* = -0.356*, P* < 0.001, for male group; *r* = -0.677*, P* < 0.001, for female group).

In male group, SMIs were negatively correlated with age in all muscle groups, especially for PM and PVM (*r* = -0.445, *P* < 0.001; *r* = -0.436, *P* < 0.001, respectively). In female group, SMIs were negatively correlated with age in all muscle groups except PMM, especially for RA (*r* = -0.481, *P* < 0.001) (Table 3).

**Table 3:** Correlation between musculoskeletal mass and age

Parameter	Age (male)		Age (female)	
	***r* value**	***P* value**	***r *value**	***P* value**
vBMD	-0.356	< 0.001	-0.677	< 0.001
AWM SMI	-0.300	< 0.001	-0.333	< 0.001
RA SMI	-0.360	< 0.001	-0.481	< 0.001
PM SMI	-0.445	< 0.001	-0.153	0.012
PVM SMI	-0.436	< 0.001	-0.194	0.001
PMM SMI	-0.258	0.001	0.005	0.940

*AWM* abdominal wall muscles, *PM* paravertebral muscles, *PMM* psoas major muscle, *PVM* posterior vertebral muscles, *RA* rectus abdominis, *SMI* skeletal muscular mass index, *vBMD* volumetric bone mineral density

### Comparison of muscular mass among varying bone mass

For both genders, SMIs decreased continuously with reduced vBMD in all muscle groups except for PM and PMM. In male group, SMIs of RA and PM in osteopenia group were significantly lower than those in the normal group (*P* = 0.001 and 0.023, respectively). SMIs of AWM and PVM in osteoporosis group were significantly lower than those in the normal group (*P* = 0.018 and 0.028, respectively). In female group, only SMI of RA in osteopenia group was significantly lower than that in the normal group (*P* = 0.007), and only SMI of RA in osteoporosis group was significantly lower than that in the osteopenia group (*P* < 0.001). SMIs of AWM and PVM in osteoporosis group was significantly lower than those in the normal group (*P* = 0.004 and 0.023, respectively) (Table 4).

**Table 4:** Comparison of muscular mass among varying bone mass

**SMI (cm**^**2**^**/m**^**2**^**)**	**Bone mass (male)**				**Bone mass (female)**			
	**Nomal**	**Osteopenia**	**Osteoporosis**	***P* value**	**Nomal**	**Osteopenia**	**Osteoporosis**	***P* value**
	***n*=88**	***n*=60**	***n*=14**		***n*=91**	***n*=95**	***n*=82**	
AWM	20.83±4.46	19.56±3.41	17.62±3.43^a^	0.011	15.22(13.46,17.48)	14.51(13.12,16.00)	12.93(10.92,14.56)^a^	0.014
RA	4.21±1.57	3.35±1.19^a^	3.00±1.13^a^	<0.001	3.04±0.99	2.62±0.82^a^	1.96±0.97^ab^	<0.001
PM	28.91±3.92	27.18±3.78^a^	27.67±3.36	0.025	22.86±2.73	22.25±3.05	22.46±3.85	0.425
PVM	16.66±2.39	15.88±2.35	14.81±3.14^a^	0.015	13.86±1.93	13.48±2.14	12.76±2.52^a^	0.005
PMM	8.67±1.8	8.50±1.78	10.01±2.47^ab^	0.023	6.48(5.45,7.62)	6.40(5.40,7.17)	7.04(5.42,10.46)^a^	0.004

Data were expressed as mean ± standard deviation or median (interquartile range)

*AWM* abdominal wall muscles, *PM* paravertebral muscles, *PMM* psoas major muscle, *PVM* posterior vertebral muscles, *RA* rectus abdominis, *SMI* skeletal muscular mass index

^a^Compared with normal group, *P*< 0.05

^b^Compared with osteopenia group, *P*< 0.05

### Correlation between muscular mass and bone mass

In male group, SMIs were positively correlated with vBMD in all muscle groups except PMM, especially for RA (*r* = 0.309, *P* < 0.001; *r* = 0.208, *P* = 0.008 after controlling for age).

In female group, SMIs were positively correlated with vBMD in all muscle groups except PM and PMM, especially for RA (*r* = 0.444, *P* < 0.001; *r* = 0.217, *P* < 0.001 after controlling for age) (Table 5).

**Table 5:** Correlation between muscular mass and bone mass

SMI	vBMD (male)				vBMD (female)			
	***r* value**	***P* value**	***r***^***a***^** value**	***P***^***a***^** value**	***r* value**	***P* value**	***r***^***a***^** value**	***P***^***a***^** value**
AWM	0.273	< 0.001	0.188	0.017	0.402	< 0.001	0.251	< 0.001
RA	0.309	< 0.001	0.208	0.008	0.444	< 0.001	0.217	< 0.001
PM	0.212	0.007	0.064	0.419	0.066	0.284	-0.016	0.795
PVM	0.269	0.001	0.135	0.088	0.206	0.001	0.085	0.165
PMM	-0.100	0.205	-0.178	0.024	-0.109	0.075	-0.164	0.007

*AWM* abdominal wall muscles, *PM* paravertebral muscles, *PMM* psoas major muscle, *PVM* posterior vertebral muscles, *RA* rectus abdominis, *SMI* skeletal muscular mass index, *vBMD* volumetric bone mineral density

^a^ After controlling for age

### Diagnostic efficacy analysis of varying muscular mass

**Fig. 2:**
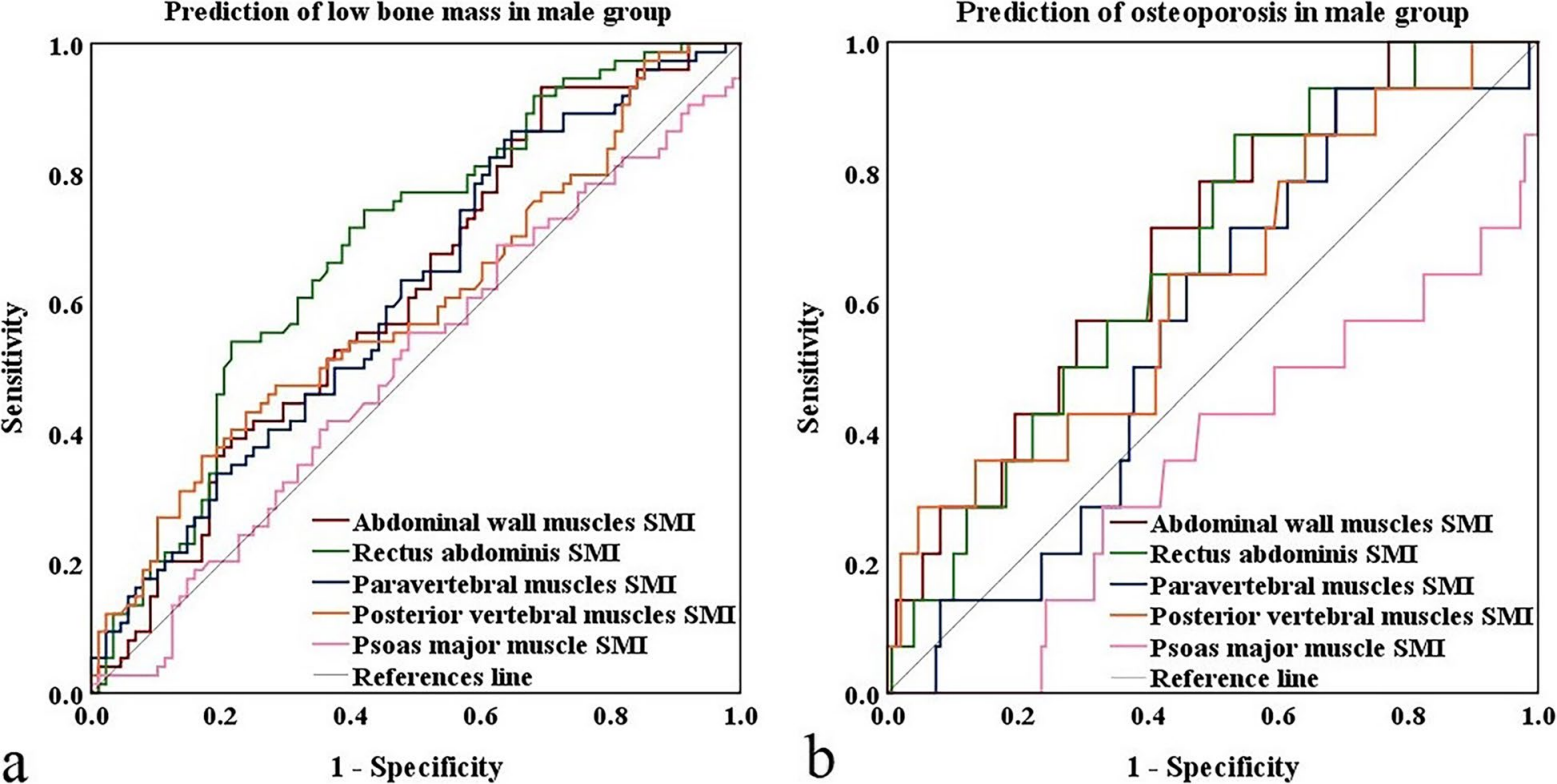
**(a)** ROC curves of SMIs of five muscles for prediction of low bone mass in male group. **(b)** ROC curves of SMIs of five muscles for prediction of osteoporosis in male group

In male group, the areas under the curves (AUCs) to differentiate between normal bone mass and low bone mass were 0.613, 0.683, 0.609, 0.594, and 0.502 for SMIs of AWM, RA, PM, PVM, and PMM, respectively. The AUCs to differentiate between non- osteoporosis and osteoporosis were 0.686, 0.667, 0.559, 0.626, and 0.356 for SMIs of AWM, RA, PM, PVM, and PMM, respectively **(**Fig. 2**)**.

**Fig. 3:**
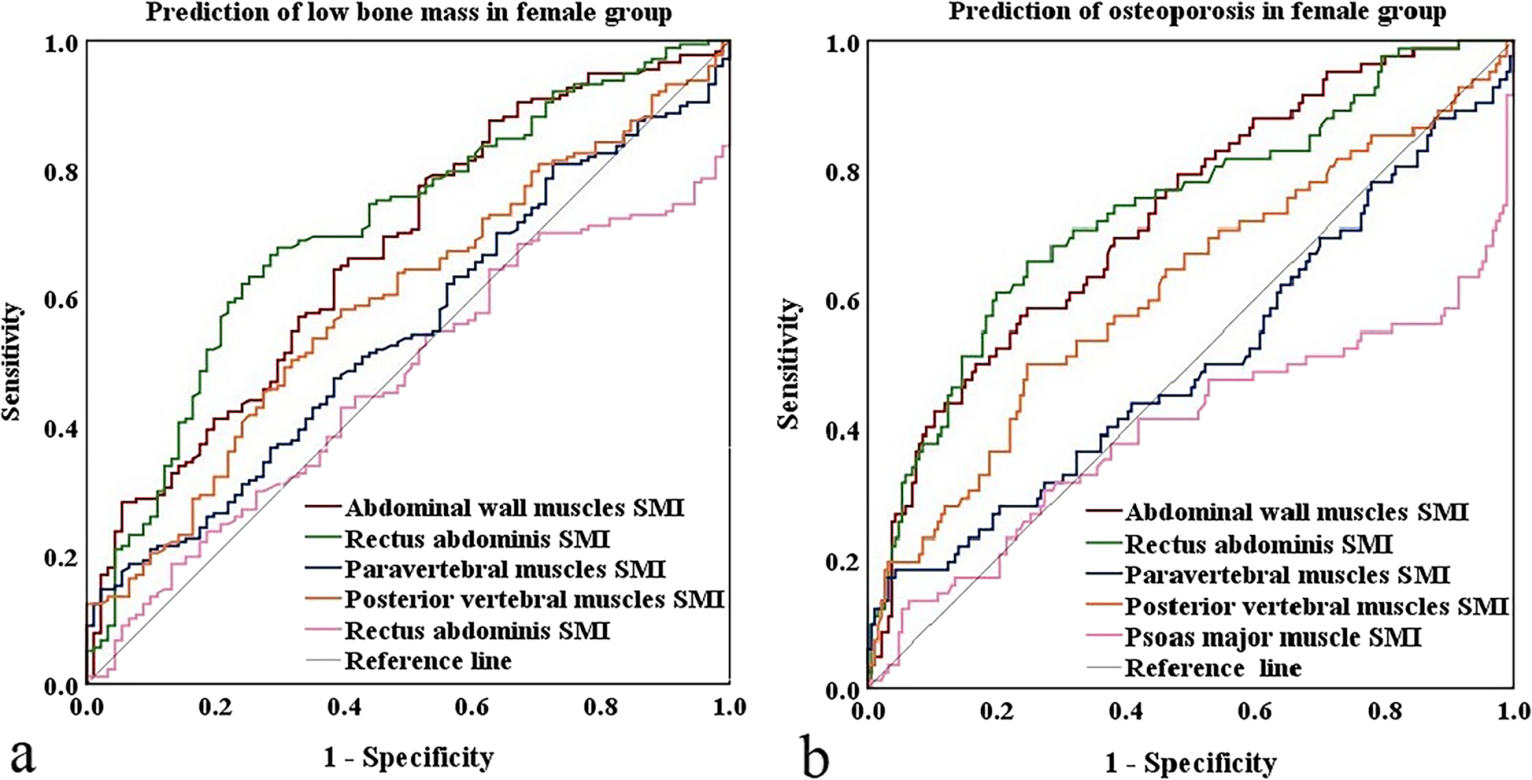
**(a)** ROC curves of SMIs of five muscles for prediction of low bone mass in female group. **(b)** ROC curves of SMIs of five muscles for prediction of osteoporosis in female group

In female group, the AUCs to differentiate between normal bone mass and low bone mass were 0.673, 0.708, 0.547, 0.595, and 0.470 for SMIs of AWM, RA, PM, PVM, and PMM, respectively. The AUCs to differentiate between non-osteoporosis and osteoporosis were 0.729, 0.737, 0.506, 0.616, and 0.398 for SMIs of AWM, RA, PM, PVM, and PMM, respectively **(**Fig. 3**)**.

The cutoff, sensitivity, specificity, positive predictive value, and negative predictive value of SMIs of five muscle groups for predicting low bone mass and osteoporosis in male and female groups were showed in Tables 6 and 7, respectively.

**Table 6:** Receiver-operating characteristic curve parameters of SMIs for prediction of low bone mass and osteoporosis in male group

SMI	*P* value	AUC (95% CI)	Cutoff (cm^2^/m^2^)	Sensitivity (%)	Specificity (%)	PPV (%)	NPV (%)
AWM	0.014^a^	0.613(0.533–0.688)	23.42	93.24	30.68	53.10	84.40
	0.006^b^	0.686(0.609–0.757)	19.13	71.43	59.46	14.30	95.70
RA	< 0.001^a^	0.683(0.606–0.754)	3.07	54.05	78.41	67.80	67.00
	0.013^b^	0.667(0.589–0.739)	3.84	85.71	46.62	13.20	97.20
PM	0.013^a^	0.609(0.530–0.685)	30.41	86.49	35.23	52.90	75.60
	0.395^b^	0.559(0.479–0.637)	30.01	92.86	31.08	11.30	97.90
PVM	0.038^a^	0.594(0.514–0.670)	14.85	36.49	82.95	64.30	60.80
	0.115^b^	0.626(0.547–0.701)	12.61	28.57	95.27	36.40	93.40
PMM	0.961^a^	0.502(0.423–0.582)	6.05	2.27	89.77	18.20	52.30
	0.076^b^	0.356(0.283–0.435)	11.09	8.78	64.29	72.20	6.30

*AUC* area under the curve, *AWM* abdominal wall muscles, *CI* confidence interval, *NPV* negative predictive value, *PM* paravertebral muscles, *PMM* psoas major muscle, *PPV* positive predictive value, *PVM* posterior vertebral muscles, *RA* rectus abdominis, *SMI* skeletal muscular mass index

^a^ Prediction of low bone mass

^b^ Prediction of osteoporosis

**Table 7:** Receiver-operating characteristic curve parameters of SMIs for prediction of low bone mass and osteoporosis in female group

SMI	*P* value	AUC (95% CI)	Cutoff (cm^2^/m^2^)	Sensitivity (%)	Specificity (%)	PPV (%)	NPV (%)
AWM	< 0.001^a^	0.673(0.614–0.729)	14.68	64.41	61.54	76.50	47.10
	< 0.001^b^	0.729(0.672–0.782)	13.31	58.54	75.27	51.10	80.50
RA	< 0.001^a^	0.708(0.650–0.762)	2.72	67.80	70.33	81.60	52.90
	< 0.001^b^	0.737(0.680–0.789)	2.29	65.85	75.27	54.00	83.30
PM	0.189^a^	0.547(0.486–0.608)	18.55	14.69	97.80	92.90	37.10
	0.882^b^	0.506(0.445–0.567)	18.13	18.29	95.70	65.20	72.70
PVM	0.007^a^	0.595(0.533–0.654)	13.29	58.19	60.44	74.10	42.60
	0.003^b^	0.616(0.555–0.675)	12.28	50.00	75.27	47.10	77.30
PMM	0.419^a^	0.470(0.409–0.531)	8.16	15.38	72.88	22.60	62.60
	0.018^b^	0.398(0.339–0.460)	8.55	8.60	58.54	32.00	22.00

*AUC* area under the curve, *AWM* abdominal wall muscles, *CI* confidence interval, *NPV* negative predictive value, *PM* paravertebral muscles, *PMM* psoas major muscle, *PPV* positive predictive value, *PVM* posterior vertebral muscles, *RA* rectus abdominis, *SMI* skeletal muscular mass index

^a^ Prediction of low bone mass

^b^ Prediction of osteoporosis

## Discussion

Decreased muscle mass, strength and function can significantly increase the risk of osteoporosis, while decreased bone mass can also significantly increase the prevalence of sarcopenia [6–8]. The co-existence of sarcopenia and osteoporosis, named mobility disorder syndrome, interacts with each other and makes the elderly population susceptible to falls, with high risks of disability and mortality [22].

The occurrence and development of primary osteoporosis are closely related to changes in hormones and bone marrow microenvironment [23, 24]. It is well-known that the ratio of red and yellow bone marrow continues to decrease with age, which is a critical cause for the low BMD [24, 25]. Our results also confirmed that vBMD was negatively correlated with age, especially in female whose vBMD was significantly decreased earlier than male (50–59 years vs. 60–69 years). It is clear that rapid decline in the level of oestrogen during perimenopause leads to bone loss through increased bone turnover (activation of osteoclast bone resorption and inhibition of osteoblast bone formation) [23, 26]. Most of the current studies use DXA or bio-impedance analysis (BIA) to measure appendicular skeletal muscle mass, using height squared correction to obtain the appendicular skeletal muscle mass index (ASMI). The EWGSOP, International Working Group on Sarcopenia, Asian Working Group for Sarcopenia have slightly different thresholds, generally using either two SDs below the mean levels of young healthy adults or the lowest quintile of ASMI as the threshold for sarcopenia [13]. In 2019, the EWGSOP suggested that low muscle mass was defined as an ASMI < 5.5 kg/m^2^ in women and ASMI < 7.0 kg/m^2^ in men [4]. In recent years, QCT has been increasingly used to assess the area of total muscles at the level of L3 after adjustment for height squared as SMI for the reference standard of sarcopenia. A meta-analysis showed that the most common cut-off values of normal SMIs ranged from 52 to 55 cm^2^/m^2^ for men and from 39 to 41 cm^2^/m^2^ for women on QCT [10]. In our study, the proportion of normal SMIs for men was between 16.67% (SMI ≥ 55 cm^2^/m^2^) and 24.07% (SMI ≥ 52 cm^2^/m^2^) and for women between 18.28% (SMI ≥ 41 cm^2^/m^2^) and 28.73% (SMI ≥ 39 cm^2^/m^2^) according to the above criteria [10].

Sarcopenia is an age-related systemic disease with multiple mechanisms involved [27]. The diagnosis of sarcopenia relies on the assessment of muscle strength and muscular mass. In this study, SMI was used as an index of muscular mass, which was found a continuous decreasing trend with age for both genders. It should be noted that the muscular mass was significantly decreased in the same age range (50–59 years) for both genders, but the muscles which had changed significantly and better negative correlation between degeneration degree and age in male and female groups were not the same [(PM and PVM) vs. RA]. This phenomenon implies that there may be differences in the order of the degeneration of lumbar and abdominal muscles in different genders. Degeneration may occur first in PM and PVM in male population, while RA in female population.

There is growing evidence of an interrelationship between low BMD and sarcopenia [6–8]. Yoshimura et al. [6] indicated that osteoporosis increased the short-term risk of sarcopenia after a 4-year follow-up. Petermann-Rocha et al. [7] identified that pre‐sarcopenia was associated with 1.3‐times higher risk of osteoporosis in men, and sarcopenia was associated with 1.66‐times increased osteoporosis risk in women, compared with people without sarcopenia or pre‐sarcopenia after 7.4 years follow‐up of 168,682 participants in UK. Similarly, a study found that postmenopausal women with sarcopenia had a 12.9-fold increased risk of osteoporosis compared to non-sarcopenic ones [8].

Previous studies frequently used the muscular mass of total muscles, PM, PMM, or PVM at the level of L3 alone as an evaluation index for investigating the correlation between muscular mass and bone mass [14–18]. Kim et al. [17] demonstrated that the muscle area of PM decreased and intramuscular fat infiltration increased in postmenopausal women with compression fracture. Kajiki et al. [18] reported that SMI of PMM was positively correlated with aBMD in the entire lumbar spine and femoral neck (*r* = 0.413 and 0.525, respectively). However, the relationship between multiple muscles at the same level and bone mass was not clear. In our study, SMIs decreased continuously with reduced vBMD in all muscle groups except for PM and PMM for both genders. However, for osteopenia group, SMIs of RA and PM in male group and SMI of RA in female group were more sensitive than others to show degeneration compared with those in the normal group. RA is located on both sides of the midline of the anterior abdominal wall, whose contraction can make the spine forward flexion, lateral flexion and pelvic tilt [28]. PM, which includes the PMM, quadratus lumborum and PVM, is closely related to the spinal space position and function, and play an important role in maintaining spinal stability, balance and mobility [29]. Also, our study found that most SMIs of lumbar and abdominal muscles were positively correlated with vBMD, especially for RA in both genders. This finding implies that the changes of muscular masses of different muscles in the lumbar and abdominal region in patients with varying bone mass are asynchronous. SMI of RA may be the most sensitive marker to reflect the early degeneration of lumbar and abdominal muscles during the development of osteoporosis.

In recent years, a few reports studied the capacity of the muscular mass to diagnose osteoporosis [18, 30]. Hayashi et al. [30] indicated a positive correlation between ASMI measured by BIA and aBMD of the lumbar spine and the femur neck ( *r* = 0.44 and 0.52, respectively) in patients with chronic liver disease. Also, this research reported that the AUCs of the ASMI for predicting osteoporosis were 0.768 and 0.718 in male and female patients, respectively [30]. Kajiki et al. [18] revealed that SMI of PMM has moderate accuracy (AUC = 0.739) in predicting osteoporosis based on DXA criterion in 87 patients with degenerative spinal diseases. In our study, the more valuable muscular masses for predicting low bone mass and osteoporosis were both SMIs of AWM and RA in both genders. However, SMI of PMM in this study did not show as good diagnostic efficacy as stated in the previous report [20]. Differences in the selection and number (87 cases vs. 430 cases) of patients and the measurement method of bone mass (DXA vs. QCT) may have contributed to the apparent differences in results. It should be noted that the AUCs of SMIs of AWM and RA for predicting low bone mass and osteoporosis in both genders were very close. In our study, AWM consists of RA, internal and external abdominal oblique muscles, and transversus abdominis muscle. Therefore, SMI of RA may play a key role in SMI of AWM predicting abnormal bone mass.

There are several limitations in our study. Firstly, the number of subjects is relatively small, and all participants were recruited from a single center. Secondly, the age distribution of the subjects was uneven. Thirdly, the number of male subjects is significantly less than female, especially male with osteoporosis is relatively few, which may lead to bias. Finally, this study lacks validation to demonstrate the clinical value of the SMIs of lumbar and abdominal muscles in predicting low bone mass and osteoporosis. Therefore, a larger sample and multicenter study is required to validate our study in the future.

In conclusion, musculoskeletal mass in lumbar and abdominal region tends to decrease with age in middle-aged and elderly people. The changes of SMIs of the lumbar and abdominal muscles in patients with varying bone mass are asynchronous. SMI of RA is expected to be a promising imaging marker for predicting abnormal bone mass.

## Data Availability

The datasets generated and/or analyzed during the current study are not publicly available due to patients’ confidentiality but a coded copy of the dataset is available to all public upon request to the corresponding author.

## Abbreviations

aBMD: Areal bone mineral density
ASMI: Appendicular skeletal muscle mass index
AUC: Area under the curve
AWM: Abdominal wall muscles
BIA: Bio-impedance analysis
CSA: Cross-sectional area
DXA: Dual X-ray absorbtiometry
EWGSOP: European Working Group on Sarcopenia in Older People
ICC: Intraclass correlation coefficient
L3: Lumbar 3
PM: Paravertebral muscles
PMM: Psoas major muscle
PVM: Posterior vertebral muscles
QCT: Quantitative computer tomography
RA: Rectus abdominis
ROC: Receiver-operating characteristic
ROI: Regions of interest
SD: Standard deviation
SMI: Skeletal muscular mass index
vBMD: Volumetric bone mineral density

## References

[1] Cruz-Jentoft AJ, Baeyens JP, Bauer JM, Boirie Y, Cederholm T, Landi F (2010). Sarcopenia: European consensus on definition and diagnosis: Report of the European Working Group on Sarcopenia in Older People. Age Ageing.

[2] Petermann-Rocha F, Balntzi V, Gray SR, Lara J, Ho FK, Pell JP (2022). Global prevalence of sarcopenia and severe sarcopenia: a systematic review and meta-analysis. J Cachexia Sarcopenia Muscle.

[3] Salari N, Ghasemi H, Mohammadi L, Behzadi MH, Rabieenia E, Shohaimi S (2021). The global prevalence of osteoporosis in the world: a comprehensive systematic review and meta-analysis. J Orthop Surg Res.

[4] Cruz-Jentoft AJ, Bahat G, Bauer J, Boirie Y, Bruyère O, Cederholm T (2019). Sarcopenia: revised European consensus on definition and diagnosis. Age Ageing.

[5] NIH Consensus Development Panel on Osteoporosis Prevention, Diagnosis, and Therapy (2001). Osteoporosis prevention, diagnosis, and therapy. JAMA.

[6] Yoshimura N, Muraki S, Oka H, Iidaka T, Kodama R, Kawaguchi H (2017). Is osteoporosis a predictor for future sarcopenia or vice versa? Four-year observations between the second and third ROAD study surveys. Osteoporos Int.

[7] Petermann-Rocha F, Ferguson LD, Gray SR, Rodríguez-Gómez I, Sattar N, Siebert S (2021). Association of sarcopenia with incident osteoporosis: a prospective study of 168,682 UK biobank participants. J Cachexia Sarcopenia Muscle.

[8] Sjöblom S, Suuronen J, Rikkonen T, Honkanen R, Kröger H, Sirola J (2013). Relationship between postmenopausal osteoporosis and the components of clinical sarcopenia. Maturitas.

[9] Kim KM, Jang HC, Lim S (2016). Differences among skeletal muscle mass indices derived from height-, weight-, and body mass index-adjusted models in assessing sarcopenia. Korean J Intern Med.

[10] Amini B, Boyle SP, Boutin RD, Lenchik L (2019). Approaches to Assessment of Muscle Mass and Myosteatosis on Computed Tomography: A Systematic Review. J Gerontol A Biol Sci Med Sci.

[11] Albano D, Messina C, Vitale J, Sconfienza LM (2020). Imaging of sarcopenia: old evidence and new insights. Eur Radiol.

[12] Yuan Y, Zhang P, Tian W, Deng X, Yue R, Ge X (2021). Application of bone turnover markers and DXA and QCT in an elderly Chinese male population. Ann Palliat Med.

[13] Li N, Li XM, Xu L, Sun WJ, Cheng XG, Tian W (2013). Comparison of QCT and DXA: Osteoporosis Detection Rates in Postmenopausal Women. Int J Endocrinol.

[14] Prado CM, Lieffers JR, McCargar LJ, Reiman T, Sawyer MB, Martin L (2008). Prevalence and clinical implications of sarcopenic obesity in patients with solid tumours of the respiratory and gastrointestinal tracts: a population-based study. Lancet Oncol.

[15] Seo HS, Lee H, Kim S, Lee SK, Lee KY, Kim NH (2021). Paravertebral Muscles as Indexes of Sarcopenia and Sarcopenic Obesity: Comparison With Imaging and Muscle Function Indexes and Impact on Cardiovascular and Metabolic Disorders. AJR Am J Roentgenol.

[16] Fortin M, Gibbons LE, Videman T, Battié MC (2015). Do variations in paraspinal muscle morphology and composition predict low back pain in men?. Scand J Med Sci Sports.

[17] Kim JY, Chae SU, Kim GD, Cha MS (2013). Changes of paraspinal muscles in postmenopausal osteoporotic spinal compression fractures: magnetic resonance imaging study. J Bone Metab.

[18] Kajiki Y, Tsuji H, Misawa H, Nakahara R, Tetsunaga T, Yamane K (2022). Psoas muscle index predicts osteoporosis and fracture risk in individuals with degenerative spinal disease. Nutrition.

[19] American College of Radiology (2018) ACR–SPR–SSR practice parameter for the performance of musculoskeletal quantitative computed tomography (QCT). American College of Radiology, Reston. Available via https://www.acr.org/-/media/ACR/Files/Practice-Parameters/QCT.pdf?la=en. Accessed 7 Nov 2018.

[20] Ishida Y, Maeda K, Yamanaka Y, Matsuyama R, Kato R, Yamaguchi M (2020). Formula for the Cross-Sectional Area of the Muscles of the Third Lumbar Vertebra Level from the Twelfth Thoracic Vertebra Level Slice on Computed Tomography. Geriatrics (Basel).

[21] Murray TÉ, Williams D, Lee MJ (2017). Osteoporosis, obesity, and sarcopenia on abdominal CT: a review of epidemiology, diagnostic criteria, and management strategies for the reporting radiologist. Abdom Radiol (NY).

[22] Binkley N, Krueger D, Buehring B (2013). What's in a name revisited: should osteoporosis and sarcopenia be considered components of "dysmobility syndrome?". Osteoporos Int.

[23] Emmanuelle NE, Marie-Cécile V, Florence T, Jean-François A, Françoise L, Coralie F (2021). Critical Role of Estrogens on Bone Homeostasis in Both Male and Female: From Physiology to Medical Implications. Int J Mol Sci.

[24] Tencerova M, Kassem M (2016). The Bone Marrow-Derived Stromal Cells: Commitment and Regulation of Adipogenesis. Front Endocrinol (Lausanne).

[25] Li Z, Hardij J, Bagchi DP, Scheller EL, MacDougald OA (2018). Development, regulation, metabolism and function of bone marrow adipose tissues. Bone.

[26] Sipilä S, Törmäkangas T, Sillanpää E, Aukee P, Kujala UM, Kovanen V (2020). Muscle and bone mass in middle-aged women: role of menopausal status and physical activity. J Cachexia Sarcopenia Muscle.

[27] Dhillon RJ, Hasni S (2017). Pathogenesis and Management of Sarcopenia. Clin Geriatr Med.

[28] Escamilla RF, Lewis C, Pecson A, Imamura R, Andrews JR (2016). Muscle Activation Among Supine, Prone, and Side Position Exercises With and Without a Swiss Ball. Sports Health.

[29] Vives MJ (2016). The paraspinal muscles and their role in the maintenance of global spinal alignment. Another wrinkle in an already complex problem. Spine J..

[30] Hayashi M, Abe K, Fujita M, Okai K, Takahashi A, Ohira H (2018). Association between sarcopenia and osteoporosis in chronic liver disease. Hepatol Res.

